# Associations of sleep with psychological problems and well‐being in adolescence: causality or common genetic predispositions?

**DOI:** 10.1111/jcpp.13238

**Published:** 2020-05-12

**Authors:** Marije C.M. Vermeulen, Kristiaan B. van der Heijden, Desana Kocevska, Jorien L. Treur, Charlotte Huppertz, Catharina E.M. van Beijsterveldt, Dorret I. Boomsma, Hanna Swaab, Eus J.W. Van Someren, Meike Bartels

**Affiliations:** ^1^ Department of Sleep and Cognition Netherlands Institute for Neuroscience an Institute of the Royal Netherlands Society for Arts and Sciences Amsterdam The Netherlands; ^2^ Department of Clinical Child and Adolescent Studies Institute of Education and Child Studies Leiden University Leiden The Netherlands; ^3^ Leiden Institute for Brain and Cognition Leiden University Leiden The Netherlands; ^4^ Department of Child and Adolescent Psychiatry Erasmus Medical Center Rotterdam The Netherlands; ^5^ Department of Biological Psychology Netherlands Twin Register VU University Amsterdam Amsterdam The Netherlands; ^6^ Amsterdam Public Health Research Institute Amsterdam UMC Amsterdam The Netherlands; ^7^ School of Experimental Psychology University of Bristol Bristol UK; ^8^ Department of Psychiatry, Psychotherapy and Psychosomatics Faculty of Medicine RWTH Aachen University Aachen Germany; ^9^ Amsterdam Neuroscience Amsterdam UMC Amsterdam The Netherlands; ^10^ Departments of Psychiatry and Integrative Neurophysiology Center for Neurogenomics and Cognitive Research (CNCR) Vrije Universiteit Amsterdam Amsterdam UMC Amsterdam The Netherlands

**Keywords:** Adolescence, sleep, behavioral problems, subjective well‐being, monozygotic twin design

## Abstract

**Background:**

Whereas short and problematic sleep are associated with psychological problems in adolescence, causality remains to be elucidated. This study therefore utilized the discordant monozygotic cotwin design and cross‐lagged models to investigate how short and problematic sleep affect psychological functioning.

**Methods:**

Adolescent twins (*N* = 12,803, 13–20 years, 42% male) completed questionnaires on sleep and psychological functioning repeatedly over a two‐year interval. Monozygotic twin pairs were classified as concordant or discordant for sleep duration and trouble sleeping. Resulting subgroups were compared regarding internalizing problems, externalizing problems, and subjective well‐being.

**Results:**

Cross‐sectional analyses indicated associations of worse psychological functioning with both short sleep and problematic sleep, and cross‐lagged models indicate bidirectional associations. Longitudinal analyses showed that an increase in sleep *problems* experienced selectively by one individual of an identical twin pair was accompanied by an increase of 52% in internalizing problem scores and 25% in externalizing problem scores. These changes were significantly different from the within‐subject changes in cotwins with unchanged sleep quality (respectively, 3% increase and 5% decrease). Psychological functioning did, however, not worsen with decreasing sleep *duration*.

**Conclusions:**

The findings suggest that sleep quality, rather than sleep duration, should be the primary target for prevention and intervention, with possible effect on psychological functioning in adolescents.

## Introduction

Sleep problems have consistently been associated with psychopathology in adults (Alvaro, Roberts, & Harris, 2013; Baglioni et al., 2016; Benca, Obermeyer, Thisted, & Gillin, 1992), children (Astill, Van der Heijden, Van IJzendoorn, & Van Someren, 2012; Gregory & Sadeh, 2012; Sivertsen et al., 2015), and adolescents (Brand & Kirov, 2011; Gregory & Sadeh, 2016; Shochat, Cohen‐Zion, & Tzischinsky, 2014). With a prevalence of approximately 25% (Ohayon, Roberts, Zulley, Smirne, & Priest, 2000), sleep problems are so common during adolescence that duration and quality of sleep may be targets for reducing the risk of developing emotional and behavioral problems (Sivertsen et al., 2015; Winkelman, 2020). Furthermore, sleep problems and to lesser extent sleep quantity have been found to be related to subjective well‐being (Jean‐Louis, Kripke, & Ancoli‐Israel, 2000; Kalak, Lemola, Brand, Holsboer‐Trachsler, & Grob, 2014; Nes, Roysamb, Reichborn‐Kjennerud, Tambs, & Harris, 2005; Paunio et al., 2009; Pilcher & Ott, 1998).

These associations reported in prospective studies (Pieters et al., 2015; Roberts & Duong, 2014; Roberts, Roberts, & Duong, 2008, 2009; Wong, Brower, & Zucker, 2009), longitudinal studies (Gregory & O'Connor, 2002; Kaneita et al., 2009; Shanahan, Copeland, Angold, Bondy, & Costello, 2014; Wang et al., 2016), and reviews (Sadeh, Tikotzky, & Kahn, 2014; Urrila, Paunio, Palomaki, & Marttunen, 2015) are not necessarily causal: Short sleep duration or sleep problems may either contribute to, concur with, or follow from psychological problems. Sleep and psychological problems could have common underlying causes, such as shared environmental influences or common genetic factors. The latter is referred to as genetic pleiotropy (Ligthart & Boomsma, 2012). Twin studies in adults showed that overlapping genes influencing the association between sleep disturbances and anxiety, depression and externalizing behaviors (Barclay, Eley, Maughan, Rowe, & Gregory, 2011; Gasperi, Herbert, Schur, Buchwald, & Afari, 2017; Gregory, Buysse, et al., 2011). Gregory, Rijsdijk, Lau, Dahl, and Eley (2009) found that sleep problems at the age of 8 years predicted depression at age 10 and suggested that this association was largely due to genetic influences. The same pattern of genetic overlap has been reported for well‐being (Nes et al. 2005). They suggested that genetic factors favoring subjective well‐being also protect against sleep problems. A cross‐sectional study (Barnes & Meldrum, 2015) among identical adolescent twins also showed overlapping genetic influences for sleep duration and adolescent developmental problems. After adjustment for genetic and shared environmental influences (since these are identical for identical twins), a shorter sleep duration remained associated with worse self‐control and depressive symptoms. However, while these findings are suggestive of a causal effect of sleep duration or quality on adolescent developmental problems, such a conclusion would require support by longitudinal data. One longitudinal study revealed that the association between short sleep duration and mental health status in monozygotic, that is, genetically identical, twin adolescents could not be attributed to shared genetic and environmental factors, thus providing additional support for a causal contribution (Matamura et al., 2014).

In order to better evaluate whether the duration and quality of sleep may causally contribute to psychological functioning, we used the powerful longitudinal discordant monozygotic (MZ) cotwin design (De Moor, Boomsma, Stubbe, Willemsen, & De Geus, 2008; Treur et al., 2015). This design can evaluate whether within monozygotic twin pairs differ and change over time with respect to sleep and psychological functioning. Because monozygotic twins are genetically identical and growing up in the same family, the design enables to rule out genetic and shared environmental influences when evaluating whether the duration and quality of sleep contributes to internalizing problems, externalizing problems and subjective well‐being. Moreover, cross‐lagged models were used to investigate the direction of effect, that is, whether sleep precedes and predicts later psychological functioning or rather the longitudinal association has a reciprocal nature.

## Methods

### Participants

Twins (*N* = 12,803, age range 13–20 year, 42% male, 4,711 MZ) of the Netherlands Twin Register (NTR) (Bartels et al., 2007; Van Beijsterveldt et al., 2013) birth cohorts 1986–1999 voluntary completed surveys including questions on psychological functioning and sleep, once or twice with an interval of about two years (overall response rate 47%, Van Beijsterveldt et al., 2013). The average age was 14.72 years (*SD* = 0.73) at the first assessment (T1: early adolescence) and 17.20 years (*SD* = 0.81) at the second (T2: late adolescence). Cross‐sectional and longitudinal reciprocal association analyses included MZ and DZ twins of whom data on sleep and psychological functioning were available for at least one time point. The subsequent cross‐sectional and longitudinal analyses within MZ twin pairs were based on subsets derived from 4,232 MZ individuals (2,116 twin pairs) out of the 4,711 participating MZ individuals. Table 1 summarizes participant characteristics and data structure. The study was approved by the Medical Ethics Review Committee of the VU University Medical Centre Amsterdam (2003/182), and written informed consent was obtained for all participants.

**Table 1:** Overview of the sample characteristics for each of the three analysis approaches

*N* = 12,803 individuals (42% male) 4,711 MZ individuals (2,148 MZ twin pairs)^a^ 8,092 DZ individuals (3,358 DZ twin pairs) **Cross‐sectional associations** including MZ & DZ individuals			
**Sleep duration**		**Sleep problems**	
**Early adolescence**	**Late adolescence**	**Early adolescence**	**Late adolescence**
9,092 individuals	7,194 individuals^b^	9,201 individuals	7,270 individuals ^b^
18% short (<8 hr)	38% short	25% present	27% present
72% average (8–9 hr)	58% average	75% absent	73% absent
10% long (>9 hr)	4% long		

**Concordant–discordant MZ twin design** **Cross‐sectional analyses within MZ *twin pairs*** (38% male) Early adolesc. 14.74 years (0.75), Late adolesc. 17.22 years (0.82)					
**Sleep duration**			**Sleep problems**		
	**Early adolescence**	**Late adolescence**		**Early adolescence**	**Late adolescence**
	1,495 pairs	1,218 pairs^c^		1,518 pairs	1231 pairs^c^
CONC short	118	284	CONC present	149	147
CONC average	920	552	CONC absent	989	771
CONC long	60	11			
DISC short—average	228	317	DISC present–absent	380	313
DISC short—long	20	24			
DISC average—long	149	50			

**Longitudinal analyses within MZ *twin pairs***					
**Sleep duration**			**Sleep problems**		
**T1 Early adolescence**	**T2 Late adolescence**		**T1 Early adolescence**	**T2 Late adolescence**	
CONC average	DISC short ‐ average	96	CONC absent	DISC present ‐ absent	79
DISC short—average	CONC short	40	DISC present ‐ absent	CONC present	34
DISC short—long	CONC short	2			
DISC average—long	CONC average	32			
CONC long	DISC short ‐ long	1			
CONC long	DISC average ‐ long	4			

The upper part presents the number of monozygotic (MZ) and dizygotic (DZ) individuals included in the cross‐sectional regression analyses. The middle part shows the number of MZ twin pairs concordant (CONC) or discordant (DISC) for sleep duration (left) and sleep problems (right). The lower part shows the number of MZ twin pairs for each of the longitudinal concordance/discordance change profiles. Because of some incomplete data, the exact number of participants can slightly differ for analyses including internalizing problems, externalizing problems, and well‐being.

^a^ The number of twin pairs does not equal twice the number of individuals since it was not necessary that self‐report data of both twins were available.

^b^ 3738 individuals (sleep duration) and 3795 individuals (sleep problems) were included at early and late adolescence.

^c^ 618 twin pairs had sleep duration data at early and late adolescence, and 633 twin pairs reported about their sleep problems at both time points.

### Instruments

*Habitual sleep duration* was assessed by asking the participants to indicate their usual sleep duration during a regular school or working week on either a 3‐point scale: 1 = *less than 8 hours per night*, 2 = *8*–*9 hours per night*, and 3 = *more than 9 hours per night* or, for the most recent assessments, using a 6‐point scale: 1 = *5 hours or less*, 2 = *6 hours*, 3 = *7 hours*, 4 = *8 hours*, 5 = *9 hours*, and 6 = *10 hours or more*. Response distributions of the two versions were comparable, allowing for rescoring of the 6‐point scale (Te Velde et al., 2013).

*Sleep problems* were assessed with the Youth Self‐Report (YSR) (Achenbach & Rescorla, 2001; Verhulst, Van der Ende, & Koot, 1997) item on experiencing trouble sleeping ‘I have trouble sleeping’. This item was shown to be valuable for screening purposes (Gregory, Cousins, et al., 2011). Responses were rated on a 3‐point Likert scale (0 = *not true*, 1 = *somewhat or sometimes true*, and 2 = *very true or often true*). Score 2 occurred only in 5% and was combined with score 1 to obtain a dichotomous indicator of sleep problems (0 = no problems and 1 = problems). One quarter of our sample reported sleep problems, comparable to population‐based prevalence estimates (Ohayon et al., 2000; Van Litsenburg, Waumans, Van den Berg, & Gemke, 2010).

*Internalizing (INT) and externalizing (EXT) emotional and behavioral problems* were quantified using the respective Youth Self‐Report (YSR) (Achenbach & Rescorla, 2001) subscale composite scores (INT range 0–62; EXT 0‐64). The YSR is a screening tool for behavioral and emotional problems in adolescents that comprise the Achenbach System of Empirically Based Assessments (ASEBA). Adolescents are asked to fill out 118 items (112 items of the 2001 version supplemented with six items for the older version of the YSR) on a 3‐point scale based on the occurrence of internalizing behaviors (e.g., anxiety and depression symptoms) and externalizing behaviors (e.g., aggressive and rule‐breaking behaviors) during the preceding 6 months: 0 if the problem item was *not true,* 1 if the item was *somewhat or sometimes true,* and 2 if it was *very true or often true.* The YSR subscale scores for internalizing and externalizing behavior have good reliability (Cronbach’s alpha .90 for both scales) and sufficient construct validity (Achenbach & Rescorla, 2001) and external validity (De Groot, Koot, & Verhulst, 1996).

*Subjective well‐being (SWB)* was quantified using a previously validated (Bartels, Cacioppo, Van Beijsterveldt, & Boomsma, 2013) latent factor score (*M* = 0, *SD* = 1) that aggregates items from the Satisfaction with Life Scale (Diener, Emmons, Larsen, & Griffin, 1985), the Subjective Happiness Scale (Lyubomirsky & Lepper, 1999), and the Cantril Ladder General Quality of Life Scale (Cantril, 1965).

### Statistical analyses

*Cross‐sectional associations* of sleep duration and of sleep problems with psychological functioning (INT, EXT, and SWB) were evaluated using linear regression analyses on all twins (MZ & DZ) assessed at early adolescence. The analyses were repeated for the partially overlapping sample assessed at late adolescence. For sleep duration, 8–9 hr was chosen as reference category and short (<8 hr) and long (>9 hr) sleep coded as two dummy variables. Analyses were adjusted for sex, within‐sample age differences, and family relatedness using the robust cluster option in STATA version 12.0 (StataCorp LLC, College Station, TX, USA).

*Longitudinal reciprocal associations* of sleep duration and sleep problems with psychological functioning (INT, EXT, and SWB) across adolescence were examined using cross‐lagged path models. These analyses enable to examine longitudinal influences while controlling for contemporaneous associations between sleep and psychological functioning and the stability of each construct over time. MZ and DZ twins were analyzed jointly, and analyses were adjusted for sex, age, and family clustering using the complex cluster option in Mplus version 7.4 (Muthén & Muthén, 2012). The default WLSMV estimator for models including binary or categorical dependent variables was used, and the DIFFTEST option was performed to obtain a chi‐square difference test. Model fit indices included chi‐square, comparative fit index (CFI ≥ 0.95), and the root mean square error of approximation index (RMSEA ≤ 0.06). Path coefficients are standardized regression coefficients (β) and indicate effect sizes, which can be considered as small (.10 ≤ β < .30), moderate (.30 ≤ β < .50), or large (β ≥ .50) (Cohen, 1988).

For subsequent cross‐sectional and longitudinal *discordance analyses,* only MZ twins (*n* = 4,232) part of a pair were included. Twin pairs were categorized as being concordant or discordant, both for sleep duration and sleep problems. Based on the reported sleep *duration*, twin pairs received one of six labels, both at T1 and T2: (a) concordant short sleep (both twins of the pair sleep < 8 hr), (b) concordant average (both twins 8–9 hr), (c) concordant long (both twins > 9 hr), (d) discordant short‐average (one twin sleeps < 8 hr and the other 8–9 hr), (e) discordant short‐long (<8 hr vs. >9 hr), and (f) discordant average‐long (8–9 hr vs. >9 hr). Based on the dichotomous sleep *problems* score, twin pairs received one of three labels, both at T1 and T2: (a) concordant present (both twins show sleep problems, (b) concordant absent (none of the twins show sleep problems, and (c) discordant present–absent (one of the twins shows sleep problems and the other not) (Table 1).

*Cross‐sectional MZ discordance analyses* were performed both at T1 and T2 using paired t‐tests to compare psychological functioning within MZ twin pairs discordant for sleep duration and within MZ twin pairs discordant for sleep problems. Cross‐sectional MZ discordance analyses exclude confounding by genetic and shared environmental influences, but the omission of longitudinal data limits inferences regarding causality.

*Longitudinal analyses* on discordant changes over time within MZ twin pairs are more powerful to evaluate a causal contribution of sleep to psychological functioning. More specifically if, at T2 compared with T1, sleep has become worse for only one of the individuals of a MZ twin pair, the causal hypothesis expects psychological functioning of this individual to worsen significantly more than changes in psychological functioning of the cotwin with preserved sleep. To analyze these changes, within‐subject difference scores were calculated by subtracting the score for psychological functioning (INT, EXT, and SWB, respectively) on T1 from the score on T2. Then, paired t‐tests were used to compare the T2‐T1 change in psychological functioning between cotwins that differed with respect to T2‐T1 worsening of sleep. Analyses were conducted with the Statistical Package for Social Sciences (SPSS) version 23.0. An alpha level of .05 (two‐sided) was used to indicate statistical significance. Standardized mean difference (*d*) indicates effect sizes for paired t‐tests (Borenstein, Hedges, Higgins, & Rothstein, 2009) and can be considered, respectively, small .20 ≤ *d* < . 50; moderate .50 ≤ *d *< .80; or large *d* ≥ .80 (Cohen, 1988).

## Results

### Sleep duration

#### Cross‐sectional associations

##### Internalizing and externalizing

Regression analyses on all MZ and DZ twins showed that both in early and late adolescence, short sleep was associated with more INT (T1: *N* = 8,932, *B = *3.50, *t* = 15.03, *p *< .001; T2: *N* = 7,082, *B* = 2.64, *t* = 13.44, *p *< .001) and more EXT (T1: *N* = 9,092, *B* = 2.24, *t* = 13.41, *p *< .001; T2: *N* = 7,194, *B* = 1.93, *t* = 14.27, *p *< .001). Moreover, in late adolescence, long sleep was associated with more EXT (*N* = 7,194, *B* = 0.85, *t* = 2.64, *p *= .008).

##### Subjective well‐being

Both in early and late adolescence, short sleep was associated with lower SWB (T1: *N* = 9,066, *B *= −0.34, *t *= −13.60, *p *< .001; T2: *N* = 7,078, *B *= −0.17, *t *= −8.00, *p *< .001). Long sleep was not significantly associated with SWB.

#### Longitudinal reciprocal associations

##### Internalizing and externalizing

**Figure 1:**
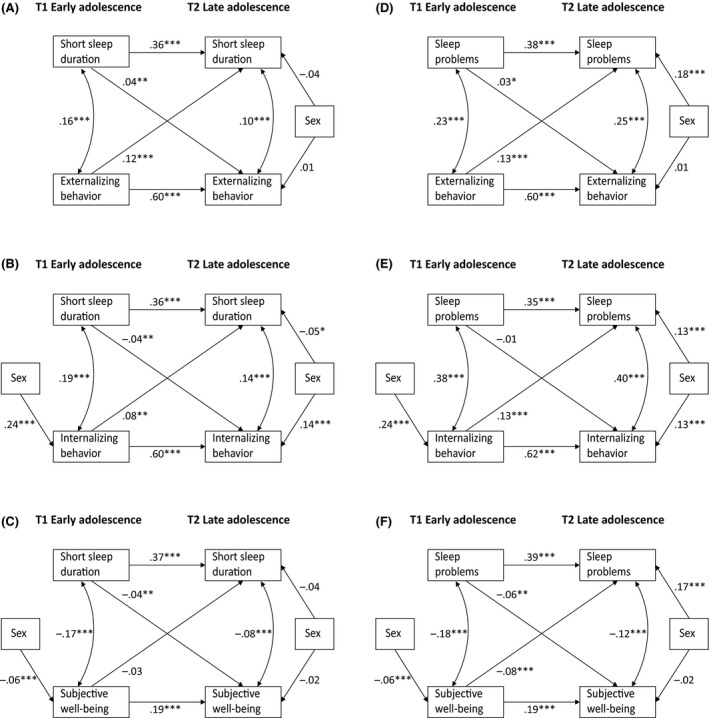
Cross‐lagged models in monozygotic and dizygotic twins from early to late adolescence within the left column reciprocal associations between short sleep duration (vs. average and long sleep duration) and externalizing behavioral problems (A), internalizing behavioral problems (B), and subjective well‐being (C). In the right column reciprocal associations between sleep problems and externalizing behavioral problems (D), internalizing behavioral problems (E), and subjective well‐being (F). All analyses (clustered by family) were adjusted for sex (0 = male, 1 = female) and age (not significant). Path coefficients are standardized regression coefficients (β) and indicate effect sizes: small (.10 ≤ β < . 30), moderate (.30 ≤ β < .50), or large (β ≥ .50). *p < .05; **p < .01; ***p < .001.

A cross‐lagged model with reciprocal associations between short sleep duration and EXT fitted the data best, χ^2^(4) = 80.91, *p *< .001, CFI = 0.96, RMSEA = 0.05. For INT, the bidirectional model with an additional path between sex and INT T1 showed a good fit, χ^2^(3) = 50.57, *p *< .001, CFI = 0.98, RMSEA = 0.04. More parsimonious models had a significantly worse model fit (Table S1). The final models were presented in Figure 1A,B.

##### Subjective well‐being

A cross‐lagged model with reciprocal associations between short sleep duration and SWB an additional path between sex and SWB T1 fitted the data best, but not all model fit indices were satisfactory, χ^2^(3) = 56.30, *p *< .001, CFI = 0.87, RMSEA = 0.04 (Table S1 and Figure 1C).

#### Cross‐sectional MZ discordance analyses

##### Internalizing and externalizing

Comparisons within the early adolescence sample of MZ twin pairs discordant for sleep duration showed that the short sleeping individuals scored higher (*t*(209) = 2.74, *p *= .007, *d *= .18) on INT (*M* = 11.50, *SD* = 8.62) than their cotwins with average sleep duration (*M* = 9.99, *SD* = 7.75). For EXT, significance was not reached (*p *= .160). In the late adolescence sample, short sleeping individuals scored significantly higher on both INT (*d *= .23) and EXT (*d *= .23) than their cotwins with average sleep duration (Table 2). No INT or EXT differences were found within the twin pairs with discordance profiles of long versus average or short sleep duration.

**Table 2:** Cross‐sectional data on psychological functioning of monozygotic twin pairs discordant for sleep duration

	*N*	Twin 1			Twin 2			*t*	*df*	*p*
		Sleep	*M*	*SD*	Sleep	*M*	*SD*			
T1 Early adolescence										
INT	210	<8 hr	11.50	8.62	8–9 hr	9.99	7.75	2.74	209	.007
	18	<8 hr	10.28	10.67	>9 hr	10.17	7.41	0.06	17	*ns*
	141	8–9 hr	7.55	6.24	>9 hr	7.92	5.99	−0.66	140	*ns*
EXT	223	<8 hr	9.71	5.95	8–9 hr	9.17	5.81	1.41	222	*ns*
	20	<8 hr	9.25	4.99	>9 hr	10.05	6.49	−0.60	19	*ns*
	146	8–9 hr	7.60	5.22	>9 hr	7.18	4.99	1.00	145	*ns*
SWB	224	<8 hr	−0.12	0.78	8–9 hr	−0.08	0.77	−0.85	223	*ns*
	20	<8 hr	−0.18	0.90	>9 hr	0.05	0.67	−1.25	19	*ns*
	148	8–9 hr	0.29	0.61	>9 hr	0.12	0.83	2.32	147	.022
T2 Late adolescence										
INT	300	<8 hr	11.61	8.53	8–9 hr	9.75	7.95	4.01	299	<.001
	23	<8 hr	10.43	8.65	>9 hr	8.74	6.49	0.73	22	*ns*
	48	8–9 hr	8.34	7.27	>9 hr	9.52	8.48	−1.32	47	*ns*
EXT	314	<8 hr	9.07	5.47	8–9 hr	7.89	4.78	3.81	313	<.001
	24	<8 hr	10.04	4.88	>9 hr	9.46	5.27	0.64	23	*ns*
	50	8–9 hr	8.62	5.38	>9 hr	8.42	4.99	0.30	49	*ns*
SWB	309	<8 hr	−0.02	0.88	8–9 hr	0.06	0.85	−1.32	308	*ns*
	24	<8 hr	0.10	0.57	>9 hr	−0.04	0.86	0.73	23	*ns*
	49	8–9 hr	0.17	0.93	>9 hr	0.14	0.88	0.22	48	*ns*

Differences in internalizing (INT) and externalizing (EXT) behavioral problems and subjective well‐being (SWB) within monozygotic twin pairs discordant for sleep duration.

##### Subjective well‐being

SWB differences were only found within the early adolescence sample of MZ twin pairs with the average‐long sleep duration discordance profile. Long sleeping individuals scored worse on SWB (*d *= .23) than their cotwins with average sleep duration (Table 2). This finding indicates that longer sleep does not necessarily accompany better psychological functioning.

#### Longitudinal analyses within MZ twin pairs

Over the interval of about two years, the sleep duration category of individuals within a MZ twin pair remained stable in 61%, decreased in 31%, and increased in 8%. Longitudinal analyses therefore focused on the effects of a decrease in sleep duration on psychological functioning in twin pairs where the discordance profile changed from (a) T1‐concordant average to T2‐discordant short‐average, (b) T1‐discordant short‐average to T2‐concordant short, and (c) T1‐discordant average‐long to T2‐concordant average. Other concordance/discordance profile changes occurred too infrequent for reliable analysis (Table 1). The analyses did not support the hypothesized causal effect of sleep duration on psychological functioning: a decrease in sleep duration experienced by only one individual of a twin pair was not accompanied by a stronger change in INT, EXT, or SWB than occurred in the genetically identical cotwin with unchanged sleep duration (Figure S1). Detailed results of the longitudinal analyses are provided in Appendix S1.

### Sleep problems

#### Cross‐sectional associations

##### Internalizing and externalizing

Regression analyses on all MZ and DZ twins showed that both in early and late adolescence, the presence of sleep problems was associated with more behavioral problems (INT T1: *N* = 9003, *B* = 5.71, *t* = 29.98, *p *< .001; INT T2: *N* = 7139, *B* = 6.34, *t* = 28.97, *p *< .001; EXT T1: *N* = 9201, *B* = 2.89, *t* = 20.42, *p *< .001; EXT T2: *N* = 7270, *B* = 2.84, *t* = 18.83, *p *< .001).

##### Subjective well‐being

Both in early and late adolescence, the presence of sleep problems was associated with lower SWB (T1: *N* = 9086, *B *= −0.34, *t *= −15.98, *p *< .001; T2: *N* = 7128, *B *= −0.28, *t *= −11.78, *p *< .001).

#### Longitudinal reciprocal associations

##### Internalizing and externalizing

A cross‐lagged model with reciprocal associations between sleep problems and EXT fitted the data best, with χ^2^(4) = 51.12, *p *< .001, CFI = 0.98, RMSEA = 0.04. For INT, the bidirectional model with an additional path between sex and INT T1 fitted the data best, χ^2^(3) = 21.40, *p *< .001, CFI = 0.99, RMSEA = 0.03. More parsimonious models worsened the model fit, see Table S2. The final models are presented in Figure 1D,E.

##### Subjective well‐being

A cross‐lagged model with reciprocal associations between sleep problems and SWB an additional path between sex and SWB T1 fitted the data best, χ^2^(3) = 26.95, *p *< .001, CFI = 0.95, RMSEA = 0.03 (Table S2 and Figure 1F).

#### Cross‐sectional MZ discordance analyses

##### Internalizing and externalizing

Both in the early and late adolescence samples of MZ twin pairs discordant for sleep problems, individuals with sleep problems scored significantly higher on INT (T1: *t*(358) = −7.48, *p *< .001, *d *= .41; T2: *t*(299) = −7.51, *p *< .001, *d *= .45) and EXT (T1: *t*(375) = −5.18, *p *< .001, *d *= .27; T2: *t*(310) = −4.69, *p *< .001, *d *= .28) than their cotwins who had no sleep problems (Table 3).

**Table 3:** Cross‐sectional data on psychological functioning of monozygotic twin pairs discordant for sleep problems

	*N*	Twin 1			Twin 2			*t*	*df*	*p*
		Sleep problem	*M*	*SD*	Sleep problem	*M*	*SD*			
T1 Early adolescence										
INT	359	Present	12.19	7.98	Absent	9.18	6.70	−7.48	358	<.001
EXT	376	Present	9.45	5.75	Absent	8.02	4.93	−5.18	375	<.001
SWB	376	Present	−0.01	0.78	Absent	0.08	0.69	2.17	375	.031
T2 Late adolescence										
INT	300	Present	13.55	8.47	Absent	10.02	7.20	−7.51	299	<.001
EXT	311	Present	9.65	5.28	Absent	8.18	5.11	−4.69	310	<.001
SWB	304	Present	−0.10	0.92	Absent	0.06	0.80	2.86	303	.005

Differences in internalizing (INT) and externalizing (EXT) behavioral problems and subjective well‐being (SWB) within monozygotic twin pairs discordant for sleep problems.

##### Subjective well‐being

Likewise, individuals with sleep problems scored significantly lower on SWB (T1: *t*(375) = 2.17, *p *= .031, *d *= .12; T2: *t*(303) = 2.86, *p *= .005, *d *= .19) than their cotwins who had no sleep problems (Table 3).

#### Longitudinal analyses within MZ twin pairs

Over the interval of about two years, sleep problems remained absent in 65%, remained present in 12%, appeared in 14%, and disappeared in 9% of the MZ twins. Longitudinal analyses focused on the effects of appearance of sleep problems on psychological functioning in twin pairs where the discordance profile changed from (a) T1‐concordant absent sleep problems to T2‐discordant present–absent and (b) T1‐discordant present–absent to T2‐concordant present sleep problems.

#### T1‐concordant absent to T2‐discordant present–absent

##### Internalizing and externalizing

**Figure 2:**
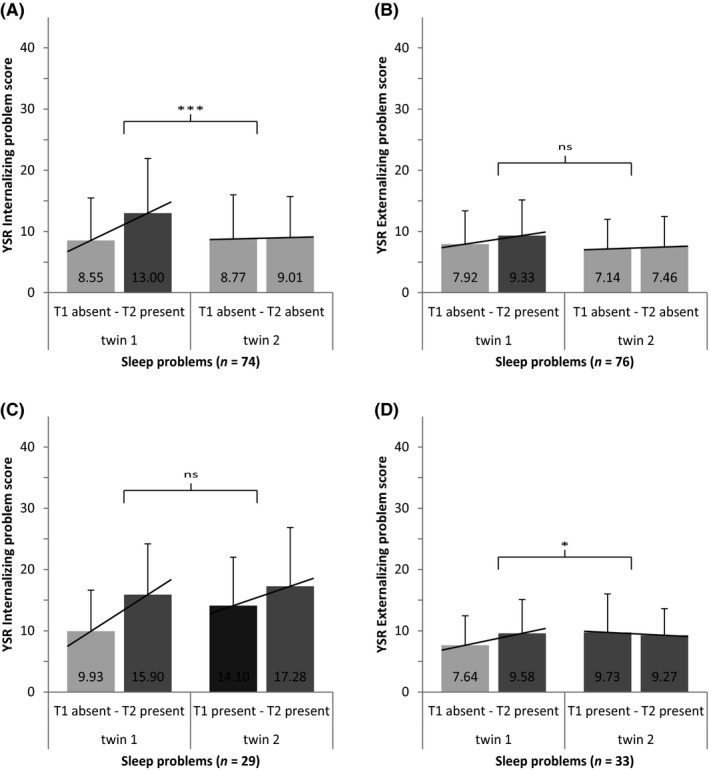
Longitudinal analyses on sleep problems and behavioral problems within monozygotic twin pairs. Within‐subject changes in Youth Self‐Report (YSR) internalizing and externalizing problems scores (+*SD*) shown for MZ twin pairs with different profiles of change in concordance/discordance for sleep problems over time: (A, B) From concordant absent (i.e., none of the twins reported sleep problems) at T1 (15 years) to discordant present–absent (i.e., one of the twins show sleep problems and the other not) at T2 (17 years); (C, D) from discordant present–absent at T1 to concordant present (i.e., both twins reported sleep problems) at T2. *p < .05 **p < .01 ***p < .001.

INT and EXT scores were comparable within twin pairs concordant for absence of sleep problems at early adolescence. Figure 2A shows that the within‐subject increase in INT from early to late adolescence was significantly larger (*t*(73) = 4.05, *p *< .001, *d *= .63) in individuals where sleep problems appeared at T2 (ΔINT = 4.45, *SD* = 7.72, 52% increase) than for their cotwins who remained without sleep problems (ΔINT = 0.24, *SD* = 5.32, 3% increase). The within‐subject increase in EXT was not significantly different (*t*(75) = 1.58, *p *= .118) for individuals where sleep problems appeared at T2 (ΔEXT = 1.41, *SD* = 5.36) as compared to their cotwins who remained without sleep problems (ΔEXT = 0.32, *SD* = 4.55) (Figure 2B).

##### Subjective well‐being

SWB scores were comparable within twin pairs concordant for absence of sleep problems at early adolescence. The within‐subject decrease in SWB was not significantly different (*t*(78) = −1.26, *p *= .211) for individuals where sleep problems appeared at T2 (ΔSWB = −0.26, *SD* = 1.18) as compared to their cotwins who remained without sleep problems (ΔSWB = −0.05, *SD* = 1.14).

#### T1‐Discordant present–absent to T2‐concordant present

##### Internalizing and externalizing

The initially higher INT (*t*(28) = −2.90, *p *= .007, *d *= .57) and EXT (*t*(32) = −2.08, *p *= .045, *d *= .37) in individuals with sleep problems at T1 as compared to their cotwins without sleep problems were no longer present at T2, when sleep problems were present in both. The within‐subject increase in INT was 5.97 (*SD* = 7.89) for individuals where sleep problems first appeared at T2 and 3.17 (*SD* = 7.78) for their cotwins who had persisting sleep problems that were present already at T1. However, this difference in increase did not reach significance (*t*(28) = 1.58, *p *= .125) (Figure 2C). The within‐subject increase in EXT was significantly larger (*t*(32) = 2.15, *p *= .039, *d *= .51) for individuals where sleep problems first appeared at T2 (ΔEXT = 1.94, *SD* = 4.96, 25% increase) compared with the small within‐subject decrease shown by their cotwins with persisting sleep problems present already at T1 (ΔEXT = −0.45, *SD* = 4.36, 5% decrease) (Figure 2D).

##### Subjective well‐being

No significant within‐pair differences were found in SWB either at T1 or T2. The within‐subject decrease in SWB of individuals where sleep problems first appeared at T2 (ΔSWB = −0.26, *SD* = 1.22) did not differ significantly (*t*(33) = −0.90, *p *= .377) from the small within‐subject decrease in the cotwins with persisting sleep problems present already at T1 (ΔSWB = −0.02, *SD* = 0.84).

## Discussion

The present study is the first to include the longitudinal discordant MZ cotwin design in combination with cross‐lagged models to investigate whether short sleep or problematic sleep may causally contribute to problems with psychological functioning during adolescence.

In line with previous prospective and longitudinal studies (Kalak et al., 2014; Roberts & Duong, 2014; Roberts et al., 2008, 2009; Sadeh et al., 2014), we found a cross‐sectional association of short sleep duration with more INT and EXT and lower SWB, all small effects. Whereas cross‐sectional findings within discordant MZ twin pairs may suggest a causal contribution of short sleep to high INT and EXT (but not to low SWB), the more powerful longitudinal analyses and the cross‐lagged models did not support such interpretation. Instead, the reciprocal association of short sleep duration and INT and EXT might be explained by overlapping genetic or shared environmental influences. This corresponds to the results of Barnes and Meldrum (2015) who found that the associations of sleep duration with many outcomes became nonsignificant after controlling for genetic and shared environmental influences.

Consistent with previous literature (Gregory & O'Connor, 2002; Kaneita et al., 2009; Pieters et al., 2015; Sadeh et al., 2014; Shanahan et al., 2014; Wang et al., 2016; Wong et al., 2009), we found that sleep problems were cross‐sectionally associated with more INT (moderate effect) and EXT (small effect) and lower SWB (small effect). In addition, both the cross‐sectional discordance analyses and powerful longitudinal analyses within MZ twin pairs are suggestive of a causal contribution of sleep problems to INT and EXT because genetic and shared environmental influences on change over time were ruled out. The results of the cross‐lagged models support this argument of a causal effect from sleep problems to behavioral problems. At the same time, the cross‐lagged models indicate that individual differences in problematic sleep and behavioral problems during adolescence have a reciprocal predictive relationship. Causal effects thus are not exclusively one‐way from sleep problems to behavioral problems. Unfortunately, we were not able to analyze the effect of behavioral problems on sleep problems with both methods, because the categorical nature of the sleep variable precluded us to utilize the longitudinal discordant monozygotic cotwin design. Our findings regarding the bidirectional effect between sleep problems and behavioral problems are consistent with the study of Wang et al. (2016). Pieters et al. (2015) on the other hand reported that sleep problems in younger adolescents predicted behavioral problems over a single year, but not the other way around. In our study, SWB appears cross‐sectionally and longitudinally associated with sleep problems, but no evidence for a causal contribution of sleep problems to SWB was found.

A recent series of behavioral and fMRI studies supports an adverse effect of sleep of poor quality rather than sleep of short duration on overnight emotion regulation (Wassing, et al., 2016; Wassing, Benjamins, Schalkwijk, & Van Someren, 2019; Wassing, Lakbila‐Kamal, et al., 2019; Wassing, Schalkwijk, et al., 2019). Two of the studies demonstrated that these effects can last for months to years. The studies moreover pinpointed a role of restless REM sleep in the adverse effects of poor quality sleep on overnight emotion regulation. Since both restless sleep and REM sleep become more prevalent at the end of the night, short sleep curtails the occurrence of restless REM sleep and may thereby actually limit the adverse effects of poor quality sleep and lead to better daytime functioning. Indeed, sleep restriction is the most effective part of the multicomponent treatment of choice for poor quality sleep: cognitive behavioral therapy for insomnia (CBTI). Long sleep is thus not better for all. Although the series of studies by Wassing et al concerns adults, unfavorable daytime effects of longer sleep have also been shown in children with an introvert and negatively affective temperament (Vermeulen, et al., 2016). Moreover, sleep extension has been shown to increase REM sleep duration particularly in adolescence (Feinberg, Davis, Bie, Grimm, & Campbell, 2012).

The lack of support for a causal contribution of sleep duration to psychological functioning is suggestive of overlapping underlying factors that explain the association. These factors could entail both overlapping genetic and shared environmental influences (Gregory & Sadeh, 2016). Previous findings suggest that especially genetic and nonshared environmental factors account for individual differences in sleep duration (Ollila et al., 2014; Te Velde et al., 2013), sleep problems (Barclay & Gregory, 2013; Palagini, Biber, & Riemann, 2014), INT, EXT, and SWB (Bartels & Boomsma, 2009; Bartels, Van de Aa, Van Beijsterveldt, Middeldorp, & Boomsma, 2011) throughout adolescence, whereas the involvement of shared environmental factors seems limited. Moreover, heterogeneity across individuals could also be due to potential gene–environment interactions. Furthermore, it should be noted that although the DNA sequence of MZ twins is identical (except for possible somatic mutations), they do not share 100% of their epigenome (Charney, 2012), which regulates gene functioning and can consequently affect behavior (Palagini et al., 2014). Epigenetic differences might contribute to differential developmental outcomes in MZ twins.

## Study evaluation and implications

The study had some limitations. Despite the unique large dataset and use of the powerful longitudinal discordant MZ cotwin design, some discordance (change) profiles occurred too infrequent to allow for reliable analysis. Notably, missing was discordance profiles of twins increasing their sleep duration to longer than 9 hr. Although this is to be expected because sleep duration decreases with age (Iglowstein, Jenni, Molinari, & Largo, 2003), as a consequence our findings concern short sleep only. Furthermore, given the origin of the NTR with survey data from a large community sample, our measures of sleep problems, sleep duration, and psychological functioning were relatively simple and limited to single items to measure trouble sleep and sleep duration. This is relatively common for large cohort studies and can be highly accurate (see e.g., Supplementary Note 1.2 of (Hammerschlag et al., 2017). Our item *Trouble Sleeping* was shown to be valuable for screening purposes (Gregory, Cousins, et al., 2011). Still, questionnaires with more questions and a continuous measure for sleep problems and sleep duration might provide higher sensitivity. Future studies could then apply a full genetically informative cross‐lagged design to dissect the etiology of the cross‐lagged links and examine the contribution of additive genes, common environment and unique environment to the longitudinal pathways. The naturalistically occurring changes in sleep duration may have heterogeneous origins especially during adolescence which is a specific developmental period during which sleep behavior and sleep physiology undergo significant maturation (Tarokh, Saletin, & Carskadon, 2016). Short sleep could indicate a discrepancy between desired and obtained sleep, but also a reduced need for sleep. This multifactorial etiology of short sleep might have contributed to the fact that we found less support from longitudinal analyses for involvement of duration than for involvement of problems of sleep in psychological functioning. Future studies may query the discrepancy between desired and obtained sleep and evaluate overlap and differences between subjectively experienced sleep and objective sleep estimates. Finally, we restricted the analyses to a sample of adolescents since we were interested in the effects of sleep on psychosocial functioning during this important developmental period. By limiting ourselves to age 14 and 16, we cannot rule out effects of childhood sleep duration, problems, or psychological functioning.

In conclusion, the present study suggests a causal contribution of problematic sleep to emotional and behavioral problems in adolescence, whereas such contribution of short sleep was not demonstrated. We moreover found support for a bidirectional association between psychological functioning and sleep problems. Nevertheless, the findings suggest that interventions that primarily target sleep problems rather sleep duration might be most effective in preventing emotional and behavioral problems in adolescents.

## Supporting information


**Appendix S1**Sleep duration: detailed results of the longitudinal analyses.
**Figure S1** **.**Longitudinal analyses on sleep duration and behavioral problems within monozygotic twin pairs.
**Table S1** **.**Fit statistics for competing cross‐lagged models on short sleep duration and psychological functioning.
**Table S2** **.**Fit statistics for competing cross‐lagged models on sleep problems and psychological functioning.
