# Supplementary material for: Associations of sleep with psychological problems and well‐being in adolescence: causality or common genetic predispositions?
Source: J Child Psychol Psychiatry. 2020 May 12;62(1):28–39. doi: 10.1111/jcpp.13238 (PMC7818180; doi:10.1111/jcpp.13238)
Supplement: Supplementary file 1 — Appendix S1Sleep duration: detailed results of the longitudinal analyses. Figure S1 .Longitudinal analyses on sleep duration and behavioral problems within monozygotic twin pairs. Table S1 .Fit statistics for competing cross‐lagged models on short sleep duration and psychological functioning. Table S2 .Fit statistics for competing cross‐lagged models on sleep problems and psychological functioning. [file JCPP-62-28-s001.docx]

**Online Supporting Information for:**

**Associations of Sleep with Psychological Problems and Well-being in Adolescence:**

**Causality or Common Genetic Predispositions?**

**Authors**

Marije C.M. Vermeulen^a,b^, PhD

Kristiaan B. van der Heijden^b,c^, PhD

Desana Kocevska^a,d^, PhD

Jorien L. Treur^e,f,g^, PhD

Charlotte Huppertz^e,f,h^, PhD

Catharina E.M. van Beijsterveldt^e,f^, PhD

Dorret I. Boomsma^e,f,i^, PhD

Hanna Swaab^b,c^, PhD

Eus J.W. van Someren* ^a,i,j^, PhD

Meike Bartels* ^e,f^, PhD

*co-senior authors

**Appendix S1. Sleep duration: detailed results of the longitudinal analyses**

To evaluate a possible causal contribution of sleep duration to psychological functioning, we compared genetically identical individuals of monozygotic twin pairs who changed with respect to their sleep duration concordance/discordance profile over a period of about two years. Outcome measures were the within-subject changes in internalizing (INT) and externalizing (EXT) behavioral problems, as well as within-subject changes in subjective wellbeing (SWB). We focused on the following three concordance/discordance profile changes that indicated a decrease in sleep duration for only one individual of the genetically identical twin pair:

***1) T1-concordant average🡪 T2-discordant short-average***

*INT & EXT*: INT and EXT scores were comparable within twin pairs concordant for 8-9hr sleep at T1. Supplemental Figures S1A and S1B show that the within-subject increase in INT and EXT from T1 to T2 was not significantly larger (INT: *t*(90)=1.57, *p*=.121; EXT: *t*(95)=1.60, *p*=.114) in individuals whose sleep duration had decreased at T2 (ΔINT=1.87, *SD*=7.73; ΔEXT=0.55, *SD*=4.90) than the increases of their co-twins who remained stable at 8-9hr (ΔINT=0.42, *SD*=5.94; ΔEXT=-0.43, *SD*=4.72).

*SWB*: SWB scores were comparable between individuals within twin pairs concordant for 8-9hr sleep at T1. The within-subject decrease in SWB from T1 to T2 in individuals whose sleep duration had decreased at T2 (ΔSWB=-0.19, *SD*=1.15) did not differ significantly (*t*(95)=-1.90, *p*=.061) from the small increase in SWB of their co-twins who remained stable at 8-9hr (ΔSWB=0.07, *SD*=0.80).

***2) T1-discordant short-average🡪 T2-concordant short***

*INT & EXT*: At early and late adolescence no significant within-pair differences were found in INT and EXT. Supplemental Figures S1C and S1D show that the within-subject increase in INT (ΔINT=2.08, *SD*=7.24) and EXT (ΔEXT=0.18, *SD*=4.64) from T1 to T2 was not significantly different (INT: *t*(37)=1.05, *p*=.299; EXT: *t*(37)=1.41, *p*=.166) for individuals whose sleep duration had decreased at T2, compared to the within-subject increase of their co-twins whose sleep duration remained less than 8hr (ΔINT=0.58, *SD*=6.90; ΔEXT=-1.18, *SD*=5.52).

*SWB*: SWB was similar within the twin pairs at early and late adolescence. The within-subject change in SWB of individuals whose sleep duration decreased at T2 (ΔSWB=0.11, *SD*=0.79) was not significantly different (*t*(39)=0.79, *p*=.436) from that of their co-twins whose sleep duration remained less than 8hr (ΔSWB=0.00, *SD*=0.93).

***3) T1-discordant average-long🡪 T2-concordant average***

*INT & EXT*: At early and late adolescence no significant within-pair differences were found in INT and EXT. The decrease in INT and EXT of individuals whose sleep duration had decreased at T2 (ΔINT=-1.28, *SD*=4.26; ΔEXT=0.07, *SD*=2.77) was not significantly different (INT: *t*(28)=1.18, *p*=.860; EXT: *t*(29)=1.47, *p*=.153) than that of their co-twins whose sleep duration remained stable at 8-9hr (ΔINT=-1.45, *SD*=5.76; ΔEXT=-1.40, *SD*=4.60) (Figures S1E and S1F).

*SWB*: At early and late adolescence no within-pair differences in SWB were found. The change in SWB of individuals whose sleep duration had decreased at T2 (ΔSWB=0.26, *SD*=0.92) was not significantly different (*t*(31)=1.96, *p*=.059) than the change in SWB of their co-twins whose sleep duration remained stable at 8-9hr (ΔSWB=-0.03, *SD*=0.52).

**Supplemental Figures**

***Figure S1*.** **Longitudinal analyses on sleep duration and behavioral problems within monozygotic twin pairs.** Within-subject changes in Youth Self Report (YSR) internalizing and externalizing problems scores (+*SD*) shown for MZ twin pairs with different profiles of change concordance/discordance for sleep duration over time: **(A-B)** From concordant average sleep (i.e., both twins reported 8-9hr sleep) at T1 (15 years) to discordant short-average (i.e., one twin sleeps <8hr and the co-twin 8-9hr) at T2 (17 years); **(C-D)** From discordant short-average at T1 to concordant short sleep (i.e., both twins sleep <8hr) at T2; **(E-F)** From discordant average-long (i.e., one twin sleeps 8-9hr and the co-twin >9hr) at T1 to concordant average sleepers at T2.

**p* < .05 ***p* < .01 ****p* < .001.

***
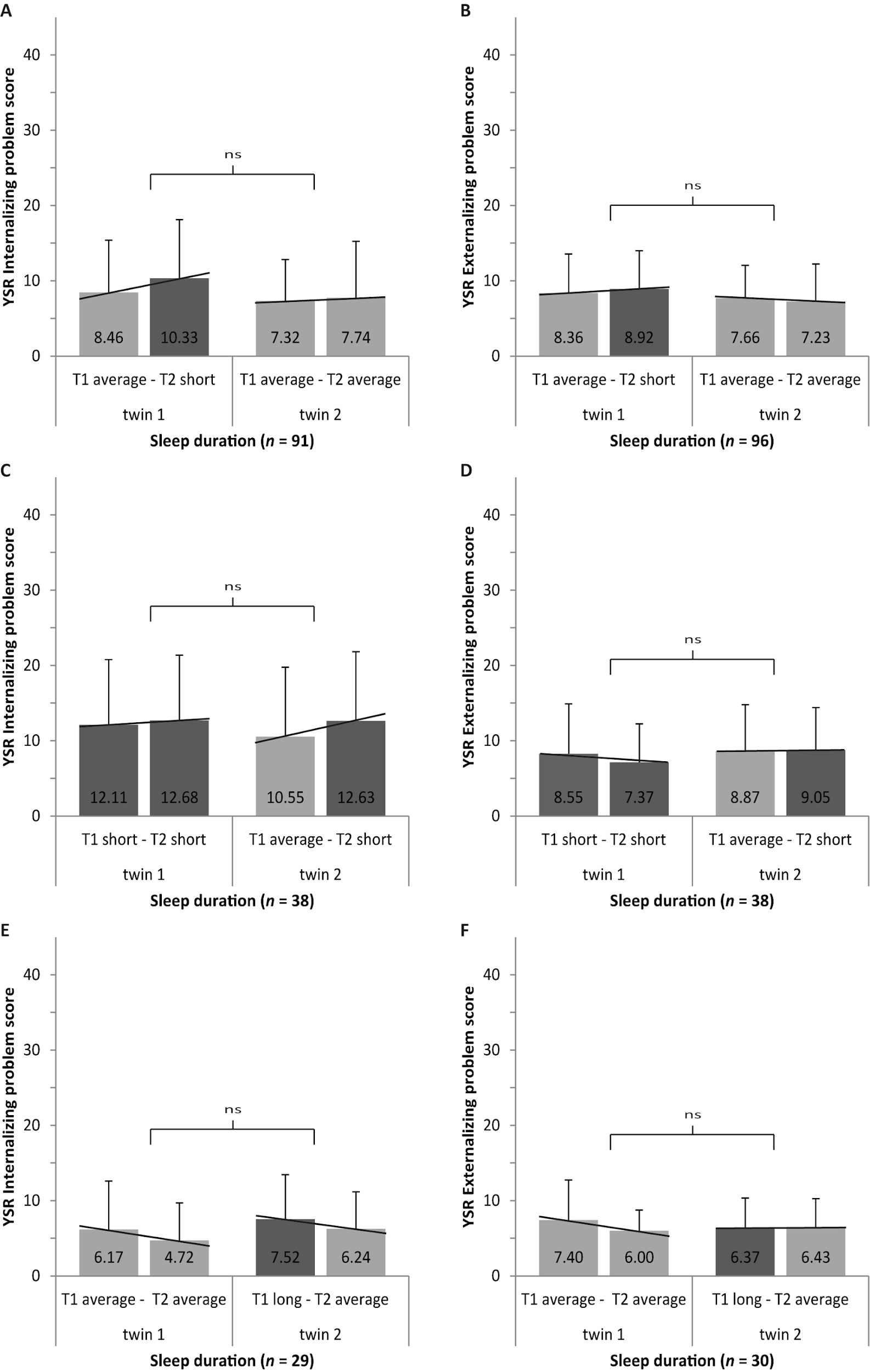
***

**Supplemental Tables**

**Table S1**

***Fit statistics for competing cross-lagged models on short sleep duration and psychological functioning.*** *The associations between short sleep duration and externalizing (EXT) and internalizing (INT) behavioral problems and subjective well-being (SWB) were examined in monozygotic and dizygotic twins.*

| **EXT** |  |  |  |  | Difference testing ^a^ | |
| --- | --- | --- | --- | --- | --- | --- |
| Model (*n* = 9297) | χ^2^(*df*) | CFI | RMSEA |  | χ^2^(*df*) | *p* |
| #1 Bidirectional | 80.91 (4) | 0.96 | 0.05 |  |  |  |
| #2 EXT 🡪 Short sleep duration | 91.65 (5) | 0.95 | 0.04 |  | 8.36 (1) | .004 |
| #3 Short sleep duration 🡪 EXT | 104.27 (5) | 0.95 | 0.05 |  | 47.96 (1) | <.001 |
| #4 Stability (no-cross-lags) | 112.98 (6) | 0.94 | 0.04 |  | 49.32 (2) | <.001 |
|  |  |  |  |  |  |  |
| **INT** |  |  |  |  | Difference testing ^a^ | |
| Model (*n* = 9264) | χ^2^(*df*) | CFI | RMSEA |  | χ^2^(*df*) | *p* |
| # Bidirectional + path sex 🡪 INT T1 | 50.57 (3) | 0.98 | 0.04 |  |  |  |
| #1 Bidirectional | 437.56 (4) | 0.80 | 0.11 |  | 409.79 (1) | <.001 |
| #2 INT 🡪 Short sleep duration | 485.92 (5) | 0.78 | 0.10 |  | 570.15 (2) | <.001 |
| #3 Short sleep duration 🡪 INT | 480.78 (5) | 0.78 | 0.10 |  | 541.97 (2) | <.001 |
| #4 Stability (no-cross-lags) | 518.25 (6) | 0.76 | 0.10 |  | 644.01 (3) | <.001 |
|  |  |  |  |  |  |  |
| **SWB** |  |  |  |  | Difference testing ^a^ | |
| Model (*n* = 9223) | χ^2^(*df*) | CFI | RMSEA |  | χ^2^(*df*) | *p* |
| # Bidirectional + path sex 🡪 SWB T1 | 56.30 (3) | 0.87 | 0.04 |  |  |  |
| #1 Bidirectional | 82.89 (4) | 0.81 | 0.05 |  | 27.29 (1) | <.001 |
| #2 SWB 🡪 Short sleep duration | 91.38 (5) | 0.79 | 0.04 |  | 37.39 (2) | <.001 |
| #3 Short sleep duration 🡪 SWB | 89.41 (5) | 0.80 | 0.04 |  | 35.38 (2) | <.001 |
| #4 Stability (no-cross-lags) | 95.68 (6) | 0.78 | 0.04 |  | 41.85 (3) | <.001 |

*Note.* CFI = comparative fit index; RMSEA = root mean square error of approximation index.
**^a^** WLSMV estimator, therefore DIFFTEST option was used to obtain a chi-square difference test. Significance indicates a worse model fit.

**Table S2**

***Fit statistics for competing cross-lagged models on sleep problems and psychological functioning.*** *The associations between sleep problems and externalizing (EXT) and internalizing (INT) behavioral problems and subjective well-being (SWB) were examined in monozygotic and dizygotic twins.*

| **EXT** |  |  |  |  | Difference testing ^a^ | |
| --- | --- | --- | --- | --- | --- | --- |
| Model (*n* = 9289) | χ^2^(*df*) | CFI | RMSEA |  | χ^2^(*df*) | *p* |
| #1 Bidirectional | 51.12 (4) | 0.98 | 0.04 |  |  |  |
| #2 EXT 🡪 Sleep problems | 57.89 (5) | 0.97 | 0.03 |  | 5.76 (1) | .016 |
| #3 Sleep problems 🡪 EXT | 62.68 (5) | 0.97 | 0.04 |  | 69.60 (1) | <.001 |
| #4 Stability (no-cross-lags) | 68.61 (6) | 0.97 | 0.03 |  | 49.39 (2) | <.001 |
|  |  |  |  |  |  |  |
| **INT** |  |  |  |  | Difference testing ^a^ | |
| Model (*n* = 9291) | χ^2^(*df*) | CFI | RMSEA |  | χ^2^(*df*) | *p* |
| # Bidirectional + path sex 🡪 INT T1 | 21.39 (3) | 0.99 | 0.03 |  |  |  |
| #1 Bidirectional | 404.50 (4) | 0.84 | 0.10 |  | 409.68 (1) | <.001 |
| #2 INT 🡪 Sleep problems | 451.68 (5) | 0.82 | 0.10 |  | 579.29 (2) | <.001 |
| #3 Sleep problems 🡪 INT | 456.68 (5) | 0.82 | 0.10 |  | 585.50 (2) | <.001 |
| #4 Stability (no-cross-lags) | 499.27 (6) | 0.80 | 0.09 |  | 716.49 (3) | <.001 |
|  |  |  |  |  |  |  |
| **SWB** |  |  |  |  | Difference testing ^a^ | |
| Model (*n* = 9297) | χ^2^(*df*) | CFI | RMSEA |  | χ^2^(*df*) | *p* |
| # Bidirectional + path sex 🡪 SWB T1 | 26.95 (3) | 0.95 | 0.02 |  |  |  |
| #1 Bidirectional | 53.37 (4) | 0.89 | 0.04 |  | 27.28 (1) | <.001 |
| #2 SWB 🡪 Sleep problems | 60.18 (5) | 0.87 | 0.03 |  | 39.67 (2) | <.001 |
| #3 Sleep problems 🡪 SWB | 63.90 (5) | 0.87 | 0.04 |  | 44.19 (2) | <.001 |
| #4 Stability (no-cross-lags) | 68.60 (6) | 0.86 | 0.03 |  | 52.22 (3) | <.001 |

*Note.* CFI = comparative fit index; RMSEA = root mean square error of approximation index.
**^a^** WLSMV estimator, therefore DIFFTEST option was used to obtain a chi-square difference test. Significance indicates a worse model fit.
